# Evidence for inflammation-mediated memory dysfunction in gastropods: putative PLA_2_ and COX inhibitors abolish long-term memory failure induced by systemic immune challenges

**DOI:** 10.1186/1471-2202-14-83

**Published:** 2013-08-06

**Authors:** Petra M Hermann, Deborah Park, Emily Beaulieu, Willem C Wildering

**Affiliations:** 1 Department of Biological Sciences, Faculty of Science, University of Calgary, Calgary, AB T2N 1N4, Canada; 2 Hotchkiss Brain Institute, Faculty of Medicine, University of Calgary, Calgary, AB T2N 4N1, Canada

**Keywords:** Oxidative stress, Mollusc, Classical conditioning, Laminarin, Phospholipase A_2_, Lipid peroxidation, Eicosanoid, *Lymnaea stagnalis*, Inflammation, Cyclooxygenase

## Abstract

**Background:**

Previous studies associate lipid peroxidation with long-term memory (LTM) failure in a gastropod model (*Lymnaea stagnalis*) of associative learning and memory. This process involves activation of Phospholipase A_2_ (PLA_2_), an enzyme mediating the release of fatty acids such as arachidonic acid that form the precursor for a variety of pro-inflammatory lipid metabolites. This study investigated the effect of biologically realistic challenges of *L. stagnalis* host defense response system on LTM function and potential involvement of PLA_2_, COX and LOX therein.

**Results:**

Systemic immune challenges by means of β-glucan laminarin injections induced elevated H_2_O_2_ release from *L. stagnalis* circulatory immune cells within 3 hrs of treatment. This effect dissipated within 24 hrs after treatment. Laminarin exposure has no direct effect on neuronal activity. Laminarin injections disrupted LTM formation if training followed within 1 hr after injection but had no behavioural impact if training started 24 hrs after treatment. Intermediate term memory was not affected by laminarin injection. Chemosensory and motor functions underpinning the feeding response involved in this learning model were not affected by laminarin injection. Laminarin’s suppression of LTM induction was reversed by treatment with aristolochic acid, a PLA_2_ inhibitor, or indomethacin, a putative COX inhibitor, but not by treatment with nordihydro-guaiaretic acid, a putative LOX inhibitor.

**Conclusions:**

A systemic immune challenge administered shortly before behavioural training impairs associative LTM function in our model that can be countered with putative inhibitors of PLA_2_ and COX, but not LOX. As such, this study establishes a mechanistic link between the state of activity of this gastropod’s innate immune system and higher order nervous system function. Our findings underwrite the rapidly expanding view of neuroinflammatory processes as a fundamental, evolutionary conserved cause of cognitive and other nervous system disorders.

## Background

The innate immune system is the first line of defense against invading pathogens and other xenobiotic substances. Its activation is characterized by a host of cellular responses including the release of pro-inflammatory compounds such as cytokines and the production of high levels of reactive oxygen and nitrogen species (ROS and RNS) [1-3]. While these compounds play critical roles in the orchestration and execution of the host defense response, their actions are not always limited to the immune system. For instance, whereas various ROS and pro-inflammatory agents play important roles in the normal physiology of the brain, imbalances in these signalling systems have been associated with detrimental effects on brain function [4,5]. Particularly, excessive (experimentally) increased levels of ROS are associated with decreased cognitive performance in both vertebrates and invertebrates [5,6], leading to the suggestion that reducing ROS levels in animals with elevated ROS may be sufficient to improve memory function [6-8].

One of the major players in the execution of the inflammatory response recruited by ROS is Phospholipase A_2_ (PLA_2_), a family of enzymes that mediate the release of free fatty acids (FFA) from the sn-2 position of membrane phospholipids [9-12]. One of the FFA’s liberated by PLA_2_ is arachidonic acid, the primary substrate for the epoxygenase (CYP-450), lipoxygenase (LOX) and the cyclooxygenase (COX) branches of the eicosanoid pathway. Together, COX, LOX and CYP-450 generate a range of primarily inflammatory modulators [13-18]. Importantly, ROS are also generated as a by-product of COX activity, thus creating a positive feedback loop that potentially can cause escalation of PLA_2_ and COX-dependent aspects of the host defense response and substantive deregulation of lipid homeostasis [15,19].

Under normal physiological conditions, the potential damage induced by ROS and other highly reactive oxidizers is controlled by the antioxidant defense system, an array of enzymatic and non-enzymatic elements [15,19]. However, under certain conditions antioxidant defense capacity of an organism may become overwhelmed by either excessive formation of ROS and other oxidizers, failing antioxidant capacity or both. Such imbalance can lead to oxidative stress. For example, pro-oxidative shifts in cellular redox status may arise as a consequence of immune system recruitment. Interestingly, age-associated accession of immune-derived oxidative stress and its impact on phospholipid metabolism are increasingly regarded as a factor in both biological and pathological aging of the nervous system [6,9,20-25]. In view of the above and building on our recent observations implicating age-associated and experimental oxidative-stress related induction of PLA_2_ activity in learning and memory impairment [6] we investigated the question whether a biologically realistic experimental provocation of the immune system also caused cognitive impairment in our model system.

In our studies we use the pond snail *Lymnaea stagnalis (L. stagnalis)* as a model system to delineate the neurobiological intracies of oxidative-stress and age-associated memory impairment [6,26,27]. In this study we utilize an established and widely used classical appetitive reward-conditioning paradigm involving chemosensory conditioning of *L. stagnalis’* feeding behaviour (i.e., “rasping”) [26,28-32]. Using this model we investigated the effects of experimental systemic challenges to the snails’ immune system on long term associative memory (LTM) formation in the absence and presence of PLA_2_, COX and LOX inhibitors.

As systemic immune challenge we delivered β-1,3 glucan laminarin intra-coelomic by means of injection. Previous work has shown that this β-glucan is a potent stimulant of circulating haemocytes, the principal cellular effectors of *L. stagnalis’* immune response [33-37]. The response repertoire of these cells includes elimination of pathogens via phagocytosis, encapsulation and the generation of various reactive oxygen and nitrogen intermediates (e.g., H_2_O_2_, NO) what is called the respiratory burst [35-39]. Our results implicate the state of activation of *L. stagnalis* host defense system to be a contributing factor in the species’ appetitive learning performance and indicate a pivotal role for PLA_2_ and COX in the process of immune-associated suppression of the species’ learning abilities.

## Results

### *In vivo* immune response to laminarin

**Figure 1:**
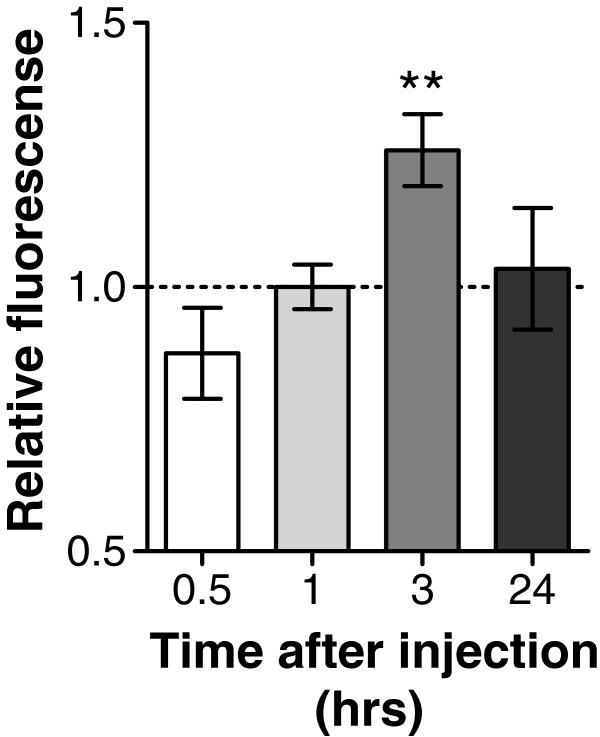
**Time dependent effect of laminarin induced release of H**_**2**_**O**_**2 **_**by haemocytes.** An increase in H_2_O_2_ production was detected when animals received a bolus injection of laminarin 3 hrs before hemocyte collection in comparison with their time corresponding vehicle treated animals. Injection of laminarin 0.5 hr, 1 hr or 24 hrs before measurement failed to detect any differences in H_2_O_2_ release between the laminarin treated groups and their time corresponding vehicle treated animals. * = p < 0.05.

To examine whether systemic delivery of a immune stimulant triggers an immune response in animals, we first performed experiments measuring haemocyte H_2_O_2_ release at varying times after intracoelomic injection of a single bolus of laminarin (~5 mg/ml haemolymph concentration) or vehicle-only. Haemocytes of both laminarin-treated and vehicle control animals were collected 0.5, 1, 3 and 24 hrs after injection and immediately submitted to fluorescent respiratory burst assays. Figure 1 shows that Amplex-Red fluorescence was very similar in samples of haemocytes collected from laminarin and vehicle treated animals 0.5 hr, 1 hr and 24 hrs after injection, but significantly differed in samples collected 3 hrs after injection (Figure 1; ANOVA interaction time × treatment; F_3,82_ = 3.04, p < 0.05 planned comparison laminarin vs. vehicle at 0.5 hr F_1,82_ = 0.578, p = 0.45; 1 hr F_1,82_ = 0.549, p = 0.46; 3 hrs F_1,82_ = 8.177, p = 0.005; 24 hrs F_1,82_ = 0.027, p = 0.86).

### Laminarin does not directly affect neuronal activity

**Figure 2:**
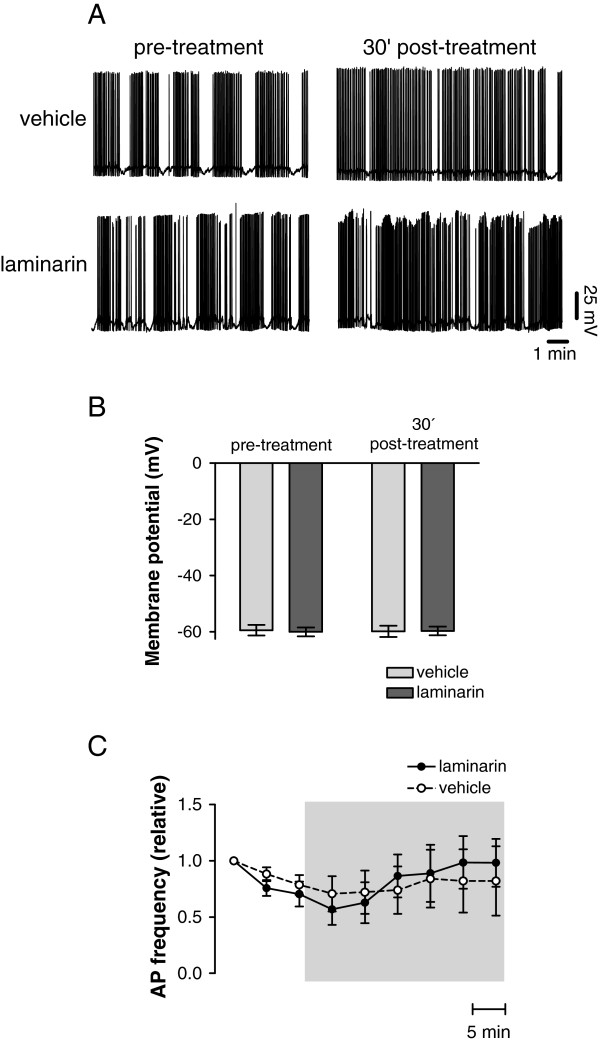
**No acute and direct effect of laminarin on neuronal activity. A**. Examples of intracellular recordings of CGCs before or after 30 minutes exposure of the isolated CNS to vehicle (saline) only (top trace) or laminarin (bottom trace). **B**. Average membrane potential of the CGCs before or 30 minutes post-treatment in vehicle only or laminarin exposed CNS. No significant difference was observed between the two treatment groups. **C**. Relative action potential frequency of CGCs 15 minutes before and during 30 minutes of exposure to laminarin or vehicle only. No significant difference was observed between the two treatment groups.

Next, we examined whether laminarin application to the nervous system directly affect neuronal activity of the cerebral giant cells (CGC), serotonergic modulatory interneurons with a critical function in the expression of appetitive LTM. CNSs were either exposed for 15 minutes to saline followed by a 30 min laminarin (5 mg/ml) treatment or to saline only for the same period of time while recording intracellular from the CGC. These experiments revealed no significant differences in CGC resting membrane potential and spontaneous action potential activity between CNS subjected to the two test conditions (Figure 2; ANOVA interaction time × treatment; F_1,9_ = 0.26, p = 0.62 for resting membrane potential and F_8,36_ = 0.44, p = 0.89 for electrical activity).

### Laminarin induced long term memory deficits

**Figure 3:**
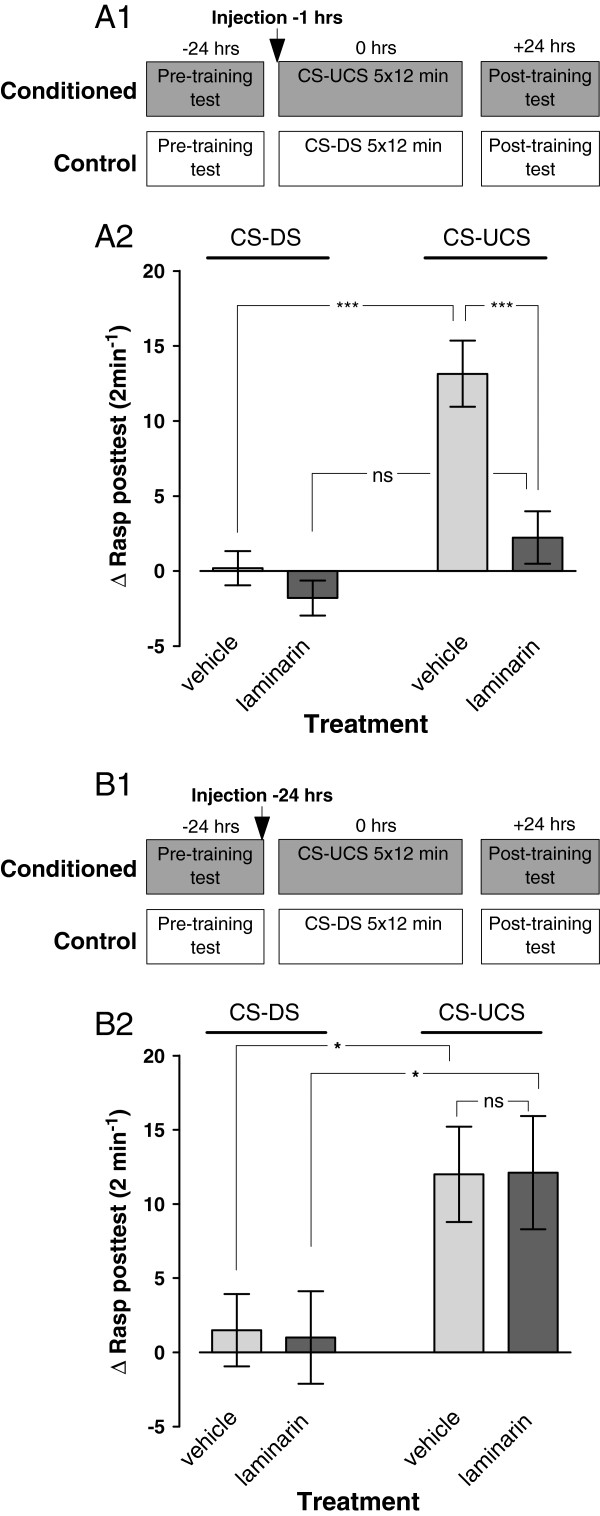
**Long-term memory assessments after laminarin injection. A1**. Protocol indicating timing of injection and start of pre- and post training tests with respect to the start of the first training session for both the conditioned (CS-UCS) and control (CS-DS) animals. **A2**. LTM assessment in animals injected 1 hr before training. There was a robust response to conditioning in vehicle injected animals. In contrast, none of the unconditioned animals or the laminarin treated conditioned animals responded with significant feeding movements in the post-training test. These results indicate LTM impairment in snails treated with laminarin 1 hr before training. **B1**. Protocol indicating timing of injection and start of pre- and post training tests with respect to the start of the first training session for both the conditioned (CS-UCS) and control (CS-DS) animals. **B2**. LTM assessment in animals treated 24 hrs before training. Both laminarin and vehicle treated conditioned animals showed a significant increase in the Δrasp values in the post-training test compared to their unconditioned peers suggesting that laminarin has no adverse effect on LTM performance when injected a day before training. * = P < 0.05, *** = p < 0.001, ns = non-significant.

To assess laminarin’s impact on the formation of appetitive long term memory (LTM) animals randomly assigned to four test groups were injected with, respectively, laminarin or vehicle-only either 1 or 24 hours before their first training session and tested for the presence of conditioned feeding responses ~24 hrs after their last training session (Figures 3A1 and 3B1). These experiments revealed a prominent suppression of conditioned feeding responses in animals that received treatment with laminarin 1 hr prior to the first training session but not in any of the other three test groups (c.f., Figures 3A2 and 3B2; ANOVA interaction training × treatment; F_1,126_ = 7.475, p = 0.007 for animals trained 1 hr after injection and ANOVA interaction training × treatment; F_1,32_ = 0.0093, p = 0.92 for animals trained 24 hrs after injection). Notably, both vehicle-injected conditioned test groups (CS-UCS) displayed robust conditioned feeding responses compared to their non-conditioned peers whether they were injected 1 hr or 24 hrs prior to their training (F_1,126_ = 26.596 p < 0.0001 and F_1,32_ = 5.476 p = 0.03, respectively). In the case of laminarin-injected animals statistically significant conditioned feeding responses were only observed in animals trained 24 hrs after they received their injections (1 hr: F_1,126_ = 3.743 p = 0.06; 24 hr F_1,32_ = 6.209, p = 0.02). While the statistic suggests near significance of the effect of behavioural conditioning in animals treated with laminarin 1 hr prior to training the results leave no doubt (see Figure 3A2) that, in contrast to the animals trained 24 hrs after treatment, the impact of training in this group is drastically reduced compared to their vehicle-injected counterparts (F_1,126_ = 22.481, p < 0.0001 for animals trained 1 hr post-treatment and F_1,32_ = 0.001, p = 0.98 for animals trained 24 hrs post-treatment).

### Laminarin does not affect chemosensory or motor functions

To assess the possibility that the behavioural results presented above were due to effects of laminarin injection on chemosensory and/or motor proficiency rather than plasticity mechanisms underlying appetitive LTM formation, we performed a number of electrophysiological and behavioural control tests.

**Figure 4:**
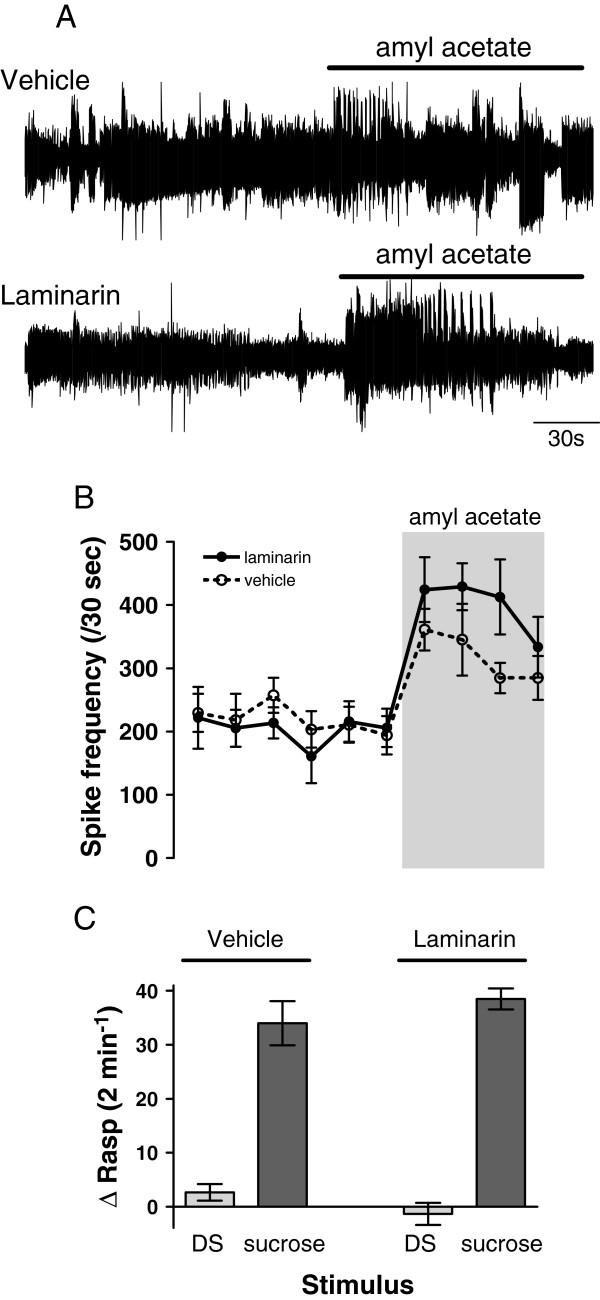
**Effect of laminarin injection on chemosensory and motor function. A**. Examples of extracellular recording of afferents in the superior lip nerve before and after application of n-amyl acetate (4 ppm) of animals injected one hour prior with vehicle only (top trace) or laminarin (bottom trace). **B**. Average action potential frequency (bin size 30 sec) of afferents in superior lip nerve 3 minutes before and 2 minutes during amyl acetate application. There is no difference in response upon n-amyl acetate application between the two different treatment groups **C**. Average change in the number of rasps evoked by application of pond water (DS) or sucrose (0.4%). Only sucrose induced a robust but similar response in animals injected with vehicle or laminarin.

First, we verified the amyl-acetate detection ability of laminarin-treated animals through analysis of evoked electrical responses recorded in the right superior lip nerve (RSLN) of semi-intact preparations. The RSLN carries chemosensory information from the lip area to the CNS. Figures 4A and 4B show representative examples of extracellular recordings of the RSLN of animals pre-treated 1 hr prior with vehicle-only and laminarin injections. The figures illustrate that robust increases in spiking frequency were recorded from the RSLN upon application of 4 ppm amyl-acetate to the lip in both experimental groups. On average, spiking frequency rapidly climbed from a baseline of around 200 events per 30 seconds immediately preceding the sensory stimulus to 300-400 events per 30 seconds during application of the stimulus in both test groups (n = 8 for both groups). Formal statistical analysis indicated a highly significant effect of peripheral amyl-acetate application on RSLN spiking frequency that was independent of prior treatment of the test animals (ANOVA time vs. treatment F_1,9_ = 1.349, p = 0.22; laminarin amyl-acetate effect in laminarin pre-treated preparations F_1,14_ = 60.87, p < 0.0001; amyl-acetate effect in vehicle-only pre-treated preparations F_1,14_ = 15.871, p < 0.001).

Behavioural assessment of the possibility that laminarin-injection adversely affects feeding motor functions was done by testing the unconditioned feeding response to sucrose of intact animals pre-treated 1 hr prior with either laminarin or vehicle-only. Figure 4C illustrates that there were no meaningful differences in the response characteristics of the two test groups. That is, the disturbance stimulus (DS) did not elicit a significant response in either of the test groups, whereas application of 0.4% sucrose induced robust, statistically indistinguishable feeding responses in animals injected with laminarin (n = 8) and animals pre-treated with vehicle-only injections (n = 8; ANOVA interaction stimulus type × treatment F_1,14_ = 2.12, p = 0.17; main effect stimulus type F_1,14_ = 148.783, p < 0.0001; sucrose response in laminarin vs. vehicle pretreated animals F_1,14_ = 0.979, p = 0.34).

### Laminarin does not affect intermediate term memory

**Figure 5:**
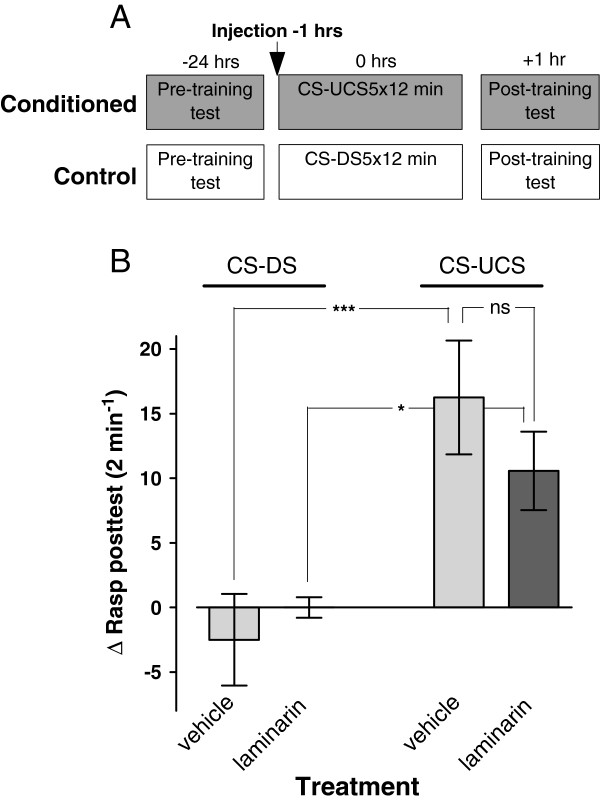
**Intermediate-term memory assessments after laminarin injection. A**. Protocol indicating timing of injection and start of pre- and post training tests with respect to the start of the first training session for both the conditioned (CS-UCS) and control (CS-DS) animals. **B**. ITM assessment in animals treated 1 hr before training. Both laminarin and vehicle treated conditioned animals showed a significant increase in the Δrasp values in the post-training test compared to their unconditioned peers suggesting that laminarin has no adverse effect on ITM performance. * = P < 0.05, *** = p < 0.001, ns = non-significant.

To test the possibility of a differential sensitivity of appetitive intermediate term memory (ITM) and LTM to systemic laminarin challenges, we examined ITM performance of vehicle and laminarin treated snails 1-2 hrs after the end of the last training trial (Figure 5A). Figure 5B shows that laminarin treatment has no effect on ITM formation (ANOVA interaction training × treatment; F_1,26_ = 1.477, p = 0.24). That is, the conditioned animals of each of the treatment groups responded significantly different from their unconditioned partners (vehicle: F_1,26_ = 16.64 p < 0.001; laminarin F_1,26_ = 4.628, p = 0.04). Conditioned vehicle and laminarin treated animals showed similar feeding responses (F_1,26_ = 1.425, p = 0.24).

### Laminarin induced LTM deficiency involves PLA_2_

**Figure 6:**
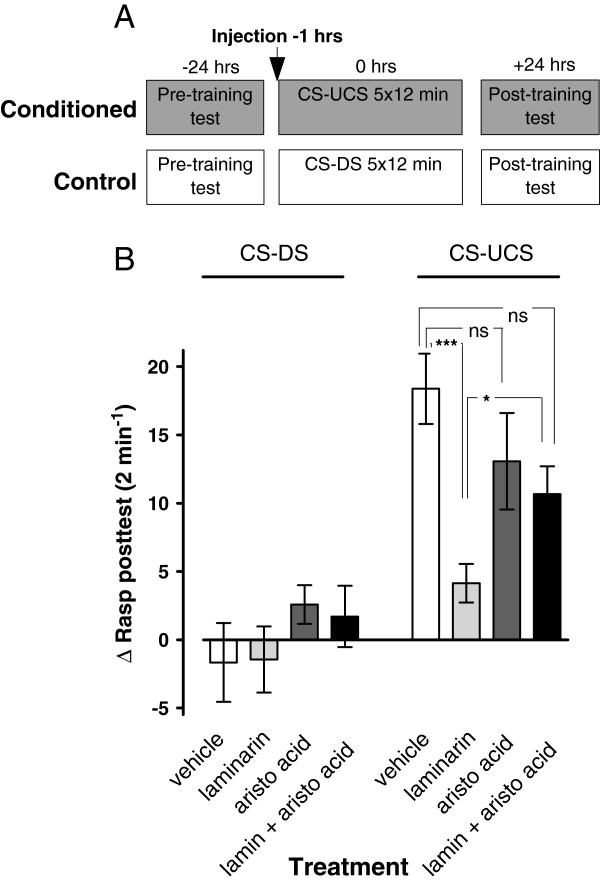
**PLA**_**2 **_**inhibition restores LTM deficiency in laminarin treated animals. A**. Protocol indicating timing of injection and start of pre- and post training tests with respect to the start of the first training session for both the conditioned (CS-UCS) and control (CS-DS) animals. **B**. LTM assessment in animals treated with either vehicle, laminarin, aristolochic acid (aristo acid) or a combined treatment of laminarin plus aristolochic acid (lamin + aristo acid). The laminarin treated conditioned group, showed a significant reduction in their Δrasp values in the post-training test compared to all other conditioned groups. Animals treated with aristolochic acid only or laminarin + aristolochic acid were not different in their conditioned response than the vehicle treated animals. This suggests that co-treatment of laminarin with a PLA_2_ inhibitor reverses the laminarin induced adverse effect on LTM performance. * = p < 0.05, *** p < 0.001, ns = not significant.

Considering PLA_2_’s pivotal role in the orchestration of key facets of the inflammatory response in many model systems, we next probed PLA_2_’s involvement in the laminarin induced LTM deficits presented above. To test this idea, four groups of animals that received, respectively, intracoelomic injections of either laminarin, aristolochic acid, laminarin + aristolochic acid or vehicle-only were behaviourally assessed as described before (Figure 6A). The results of these experiments are summarized in Figure 6B.

We found that pretreatment with the PLA_2_ inhibitor aristolochic acid counteracted laminarin’s negative effect on LTM formation (ANOVA training × laminarin-pretreatment × aristolochic acid pretreatment, F_1,135_ = 3.953, p < 0.05). Further statistical analysis indicated that this interaction resided in opposing effects of laminarin and aristolochic acid on conditioned feeding responses. That is, while pretreatment with laminarin caused a significant reduction in the conditioned feeding response compared to vehicle treated animals as before (c.f., Figure 3A2 & Figure 6B; F_1,135_ = 20.234, p < 0.0001), animals pretreated with aristolochic acid learned well (F_1,135_ = 9.934, p = 0.002) in a manner that was statistically indistinguishable from that of vehicle pretreated animals (Figure 6B c.f., white bars with dark-gray bars; F_1,135_ = 2.351, p = 0.13). Moreover, animals pretreated with the combination of laminarin and aristolochic acid did display a significant conditioned feeding response (Figure 6B black bars; F_1,135_ = 8.128, p = 0.005) that was significantly different from the laminarin injected animals (Figure 6B c.f., light gray bars with black bars; F_1,135_ = 4.937, p = 0.03).

### Laminarin induced LTM impairment is rescued by indomethacin

**Figure 7:**
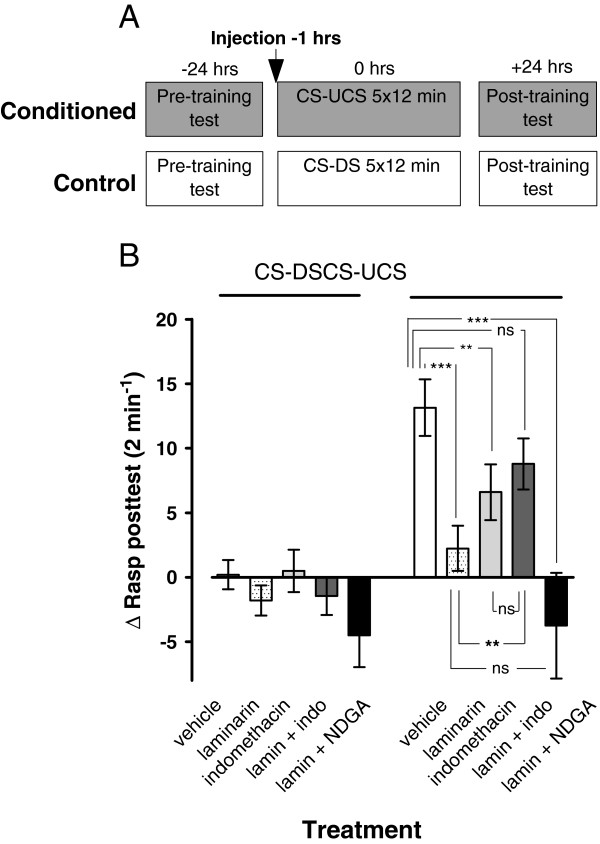
**Indomethacin restores LTM failure in laminarin treated animals. A**. Protocol indicating timing of injection and start of pre- and post training tests with respect to the start of the first training session for both the conditioned (CS-UCS) and control (CS-DS) animals. **B**. LTM assessment in animals treated with either vehicle, laminarin, indomethacin, a combined treatment of laminarin plus indomethacin (lamin + indo) or a combined treatment of laminarin plus NDGA (lamin + NDGA). None of the laminarin treated conditioned animals and laminarin + NDGA conditioned animals or any of the unconditioned animals responded with significant feeding movements upon amyl acetate application in the post-training test. Indomethacin only treated animals showed a significant increase in their Δrasp values compared to their unconditioned peers but this conditioned response was significantly smaller than observed in the vehicle conditioned animals. Both vehicle and laminarin + indomethacin treated conditioned animals responded with a significant increase in the Δrasp values compared to their unconditioned peers and the laminarin conditioned animals. This suggests that co-treatment of laminarin with a COX inhibitor reverses the laminarin induced adverse effect on LTM performance. ** = p < 0.01, *** p < 0.001, ns = not significant.

Next, we tested the possibility of the involvement of the eicosanoid pathway in the laminarin-induced LTM impairment. For this purpose we used nordihydroguaiaretic acid (NDGA) and indomethacin, broad-spectrum inhibitors of, respectively, LOX and COX, the two major branches of the eicosanoid pathway. Drug administration, training and 24 hrs LTM assessment were performed according to procedures described before (Figure 7A). Consistent with previous results, laminarin-injected animals but not vehicle-injected animals failed to show robust conditioned feeding responses 24 hrs after their last training (Figure 7B, c.f., dotted bars and white bars; ANOVA treatment x training F_4,222_ = 3.02, p = 0.019, F_1,222_ = 23.522 p < 0.0001). The black bars in Figure 7B show that concurrent treatment with laminarin and NDGA (~10 μM haemolymph concentration) did not prevent LTM failure. In fact, the amyl-acetate evoked rasping response of conditioned animals pretreated with laminarin plus NDGA was indistinguishable from their unconditioned (CS-DS) counterparts (F_1,222_ = 0.028 p = 0.87). In contrast, the conditioned feeding response of indomethacin treated animals (~10 μM haemolymph concentration) differed significantly from that of their none conditioned counterparts (Figure 7B; contrast indo conditioned vs unconditioned F_1,222_ = 4.499, p = 0.04). However, as the figure illustrates the conditioned feeding response of indomethacin treated animals was significantly attenuated when compared with conditioned vehicle treated animals (Figure 7B; contrast vehicle vs Indo F_1,222_ = 6.166, p = 0.01). Interestingly, concurrent treatment with laminarin and indomethacin significantly improved conditioned feeding responses of the trained animals. That is, in contrast to the animals treated with laminarin only, animals that received laminarin plus indomethacin showed a robust conditioned feeding response (F_1,222_ = 7.911 p = 0.005) that was neither significantly different from the conditioned response of animals treated with vehicle-only (F_1,222_ = 3.018 p = 0.09) nor from that of animals treated with indomethacin only (F_1,222_ = 0.656 p = 0.42).

### Haemocyte activation is not affected by aristolochic acid or indomethacin

**Figure 8:**
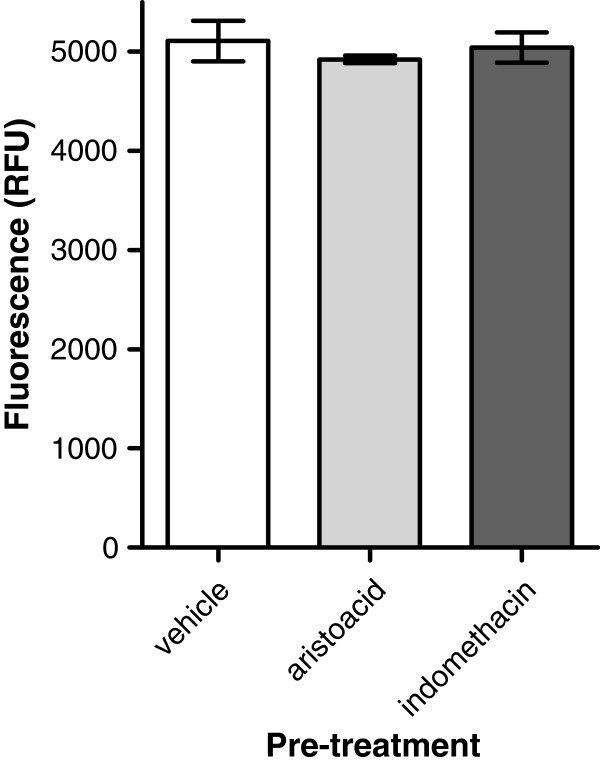
**No effect of aristolochic acid or indomethacin on laminarin induced H**_**2**_**O**_**2 **_**release by haemocytes.** Isolated haemocytes pre-incubated for 30 minutes with either aristolochic acid or indomethacin before being challenged with laminarin showed a similar H_2_O_2_ production compared to haemocytes pre-treated with vehicle only.

The effects of aristolochic acid and indomethacin described above may arise from inhibition of laminarin-induced processes at the level of immunocytes or the level of the nervous system. Thus next we explicitly tested the former possibility by measuring the impact of both compounds on laminarin induced H_2_O_2_ release by isolated haemocytes. Amplex Red fluorescence was very similar in laminarin activated haemocytes that were pre-treated with either aristolochic acid or indomethacin when compared to haemocytes pre-treated with vehicle-only (Figure 8; ANOVA; F_2,8_ = 0.401, p = 0.69).

## Discussion

The primary purpose of this study was to examine the plausibility of inflammation-mediated learning and memory dysfunction in *L. stagnalis*. The concept of inflammation and inflammation-mediated oxidative stress as agents of neural dysfunction and degeneration has become well-established in the context of mammalian nervous systems [5,7-11,17-25,40]. However, despite their prominence as neurobiological model systems of learning and memory, remarkably little is known about neuroinflammatory aspects of learning and memory dysfunction in gastropods. Recent findings implicating non-enzymatic lipid peroxidation in PLA_2_-dependent LTM failure in aging *L. stagnalis*[6,27] led us to hypothesize involvement of the snails’ immune system in learning and memory dysfunction. The current results support that hypothesis, even though many questions about the identity, modes and loci of action of the signalling processes involved remain to be answered. This conclusion hinges on the following observations: 1- systemic delivery of the immune activator laminarin induces a transient increase in H_2_O_2_ release from circulating haemocytes within 1-3 hrs after injection that dissipates within 24 hrs; 2- intracoelomic injection of laminarin 1 hr before the start of behavioural conditioning induced LTM dysfunction; 3- intracoelomic injection of laminarin 24 hrs before the start of behavioural conditioning had no impact on LTM function; 4– co-administration of laminarin with putative PLA_2_ inhibitor aristolochic acid or the putative COX inhibitor indomethacin negated laminarin’s effect on LTM; 5- Neither aristolochic acid nor indomethacin inhibited laminarin-induced haemocyte- mediated H_2_O_2_ release; 6- intracoelomic injection of laminarin did not cause obvious chemosensory or motor deficits; 7- laminarin injection did not affect intermediate term memory (ITM) function; 8- exposure of isolated CNS to laminarin did not affect electrical activity of interneuron CGC. Taken together these results support a model in which the haemocyte’s inflammatory response disrupts LTM formation by recruiting neuronal PLA_2_-mediated processes. As will be discussed below, such a model is consistent with our recent finding that PLA_2_ plays a pivotal role in LTM impairment associated with oxidative stress and old age in *L. stagnalis*[6].

### Signalling mechanisms

We propose that the inflammatory response from circulating haemocytes is a primary cause in laminarin’s behavioural effects. We base this opinion on the following evidence. First, the H_2_O_2_ release data leaves no doubt that circulating haemocytes are among the targets of laminarin in our experiments. Second, neither the chemosensory response to amyl-acetate, nor the behavioural response to sucrose application were affected by laminarin 1 hr after its injection, a time point where no measurable increase haemocyte H_2_O_2_ release had occurred yet. Third, laminarin applied to isolated CNS preparations had no effect on spontaneous spiking activity and resting membrane potential of the Cerebral Giant Cell, one of the interneurons of the feeding circuit that is crucial to the formation of appetitive LTM and that receives chemosensory synaptic input and maintains widespread synaptic connections throughout the circuit. Although it is difficult if not impossible to test all-inclusively for the absence of drug effects, these results together provide quite compelling evidence that laminarin has no substantial direct neuronal effects in at least the first 1-1.5 hr after treatment. Further investigations are required to assess whether, on the longer run, laminarin may affect components of the nervous system like perhaps the professional phagocytes known to reside in the CNS [41].

The present results show striking parallels with a previous report that aristolochic acid corrects experimental oxidative stress-induced LTM failure in *L. stagnalis* as well as age-associated LTM impairment [6]. In other words, PLA_2_ appears to lie at the root of oxidative stress-dependent, inflammation-induced and age-associated LTM impairment paradigms in the *L. stagnalis* model system. We interpret these parallels as evidence of a close causal relation between these phenomena. This interpretation resonates well with a rapidly growing literature that associates PLA_2_ with (neuro)inflammation, deregulation of lipid metabolism, and cell-, neuronal- and cognitive dysfunction in humans and other mammals [9,19-24].

We assume that aristolochic acid’s ability to restore LTM impairment in the current inflammation model reflects the drug’s actions at the level of the nervous system. This idea arises from previous evidence demonstrating that PLA_2_ inhibition with aristolochic acid reverses, within minutes, electrophysiological phenomena induced by experimental oxidative stress in isolated CNS preparations thought to contribute to LTM impairment as well as similar phenomena in the brains isolated from LTM impaired old snails [6,27]. The idea of a neural locus of aristolochic acid’s restorative actions is further supported by the present finding that the drug does not affect laminarin-induced haemocyte H_2_O_2_ release, a result consistent with prior literature emphasizing phospholipase C (PLC)/protein kinase C (PKC)-dependent signalling pathways in this process [35,36]. Parenthetically, assuming the above reasoning is true, the question of identity of the signalling intermediate(s) between haemocytes and neurons arises. There is probably more than one answer to this question. However, based on the uncanny parallels between the experimental oxidative stress model of LTM impairment that identifies non-enzymatic lipid peroxidation as a probable activator of neuronal PLA_2_[6,27] and the current data, we consider oxidizers released by activated haemocytes are plausible candidates.

At this point it is important to recognize the importance of ROS and RNS signalling in synaptic plasticity and memory formation including the induction of LTM in the very model system we used here [5,15,19,42-44]. Specifically, NO-signalling is particularly relevant during the first 5 hours of LTM consolidation in the current LTM paradigm [42,44]. Since *L. stagnalis* hemocyte’s release substantial amounts of NO together with H_2_O_2_ during their respiratory burst [35,36], the possibility that haemocyte-derived NO interferes directly with critical NO-dependent steps in appetitive LTM formation exists.

The finding that the putative COX inhibitor indomethacin, but not the putative LOX inhibitor NDGA, partially reversed laminarin’s inhibition of appetitive LTM suggests COX involvement in this process. Although this notion is consistent with the metabolic interdependence of COX and PLA_2_, this conclusion needs to be treated with some care because, as discussed in more detail below, reservation’s about indomethacin’s COX specificity. Nevertheless, the notion of COX involvement in inflammation-induced learning impairment is consistent with the enzyme’s long-standing stature as a pivotal producer of pro-inflammatory mediators [17,18,45] and not without precedent in the context of synaptic transmission and plasticity, learning and memory formation [13-16,19,46-53]. Very little, if anything, is known about inflammatory and/or neuro-modulatory implications of COX activity in *L. stagnalis*. Considering growing evidence implicating PLA_2_ and COX-metabolites in cognitive function and dysfunction in mammals [4,5,7,46-53], the current results, although not conclusive, do warrant further investigation into this matter.

### Haemocyte activation: *in vivo* immune challenge vs. *in vitro* immune challenge

Our data support the conclusion that a single intracoelomic injection of laminarin provokes an increase in haemocytic H_2_O_2_ release within 1-3 hours after injection that dissipates within 24 hrs of the injection. Whereas these results are in general agreement with those obtained in *in vitro* studies of *L. stagnalis* haemocytes, they also reveal important differences [35]. Particularly, whereas H_2_O_2_ release increases within 30 to 60 minutes after *in vitro* stimulation of isolated haemocytes with 5 mg/ml laminarin, our results indicate that the same may take up to three hours when laminarin is injected into the animal’s body cavity. Considering the much more complex biological and pharmacokinetic conditions prevailing in the whole animal, a somewhat slower and smaller *in vivo* response does not seem to be out of the ordinary. Most importantly, although we cannot pinpoint the exact time course of the response, our data do support the notion that the impact of laminarin injection on haemocyte H_2_O_2_ release is gone by the time LTM tests are performed 24 hrs after training.

### Limitations: alternative targets of laminarin and drug specificity?

As any other study relying on pharmacological interventions, our results might be affected by non-specific actions of the drugs we used. Aristolochic acid has a long track record as broad spectrum PLA_2_ inhibitor [54-56]. Other than an extensive literature on the compound’s genotoxic effects that are associated with its ability to form aristolactam-DNA adducts [57,58], we are not aware of reports of non-specific activities. It seems unlikely that DNA-adduction is a factor of importance in the present study. An observation strengthening our believe in aristolochic acid’s relative specificity is the finding that the compound does not interfere with laminarin’s ability to activate haemocytes, a process that involves a major phospholipid-dependent signalling component in the form of protein kinase C- (PKC), phospholipase C- (PLC), extracellular signal-regulated kinase- (ERK) and MAPK/ERK kinase (MEK)-dependent signalling cascade [35]. Moreover, no effect of aristolochic acid on electrophysiological, behavioural and biochemical facets were observed in *L. stagnalis* without any form of pro-oxidant present [6]. While we have no compelling reason to suspect aristolochic acid’s pharmacological activities, indomethacin warrants more extensive scrutiny. For instance, like other NSAIDs, indomethacin may modify the activity of various serine/threonine kinases [59,60]. However, electrophysiological studies on *L. stagnalis* neurons revealed no evidence of indomethacin effects on PKC nor protein kinase A (PKA) signalling at the concentrations used here [61]. Moreover, we show that indomethacin does not affect laminarin induced H_2_O_2_ release from haemocytes, which as noted before, is a PKC-dependent process. Thus, it seems unlikely that the effects of indomethacin reported here are due to interference with PKA and/or PKC signalling. However, several reports indicate that indomethacin, in addition to its ability to inhibit COX, can inhibit several types of PLA_2_ enzymes [55,62-64]. No information is available about the type of PLA_2_ underlying the current observations. Thus we cannot exclude the possibility that indomethacin’s effects reported here are in part due to the inhibition of PLA_2_. However, that uncertainty does not in any way invalidate our central conclusion that laminarin-mediated haemocyte activation induces PLA_2_-dependent LTM dysfunction.

### Differential susceptibility of ITM and LTM to systemic immune challenge

Our results show that non-aversive appetitive ITM in contrast to its LTM counterpart is not significantly compromised by systemic injection of laminarin. There are a number of noteworthy observations to make from this finding. First, it provides additional evidence that relatively short term exposure to laminarin itself does not compromise the neural systems and processes involved in the detection and execution of chemosensory-induced feeding responses. Second, it points towards independence of the neural substrates ITM and LTM and as such reiterates recent evidence by Marra et al. indicates that non-aversive appetitive ITM and LTM formation in this model follows a parallel rather than serial causal pathway [65]. Third, the current evidence that LTM is more sensitive to disruption of immune-related processes than ITM takes on a particularly intriguing perspective in view of a growing body of literature associating (neuro)inflammation with age-associated cognitive and neurological dysfunction and our own work showing LTM but not ITM to be impaired in old *L. stagnalis* in both learning and memory paradigms we use in our research [6,26].

## Conclusion

In this study we provide evidence for a role of the innate immune system in learning and memory impairment in the snail *L. stagnalis*. Specifically, our results indicate that provocation of the immune system induces a PLA_2_-dependent form of LTM dysfunction resembling the memory impairment associated with experimental (lipid per-)oxidative stress and old age in this model system. The results emphasize the significance of membrane lipid biology and enzymes of the PLA_2_ family in dysfunction of the nervous system of this invertebrate model system and underwrite the fast growing perception of these processes as a fundamental and evolutionary conserved dimension of immune- and age-associated changes in neural- and cognitive failure. As such, *L. stagnalis* provides an excellent model system for detailed fundamental investigations into the role of the innate immune system in disorders of the nervous system.

## Methods

### Animals

Snails were bred and raised under constant conditions as previously described [6,26,27,32,66]. Briefly, snails were raised in house under strictly controlled ambient conditions (light:dark 12:12, ambient temperature 18-19°C, pH 7.8-7.9). Water used in the facility was sourced from a reverse osmosis system and reconditioned to a conductivity of ~450 Ω.cm through the addition of Instant Ocean salts at 1 g/US Gallon (i.e., artificial pond water; Aquarium Systems USA). Calcium concentration was kept at saturation level (>60 mg/L as CaCO_3_) through the addition of calcium carbonate (light powder; EMD analytics, Gibbstown, New Jersey) to the tanks. In addition, snails had continuous access to sterilized cuttlefish *(Sepia officinalis)* bone (2–3 per tank). Snails were fed ad libitum with a standard diet consisting of Romaine lettuce and Aquamax-carniverous Grower 600 trout pellets (Purina Mills LLC, St. Louis, Missouri).

Health and survival characteristics of the populations were continuously monitored and evaluated using previously established methods based on the Weibull failure model [67,68]. Experimental snails were taken at random from populations with healthy demographic parameters with survival percentages >95% and chronological ages ranging between 5-7 months of age at the time of sampling (note: in our experimental populations this amounts to young sexually mature snails). Snail use and care procedures conformed to rules of the University of Calgary Animal Care and Use Policy which adheres to the guidelines, policies and standards of the Canadian Council on Animal Care (CCAC), the Canadian Association of Laboratory Animal Medicine (CALAM), standards of Veterinary Care, and the Alberta Veterinary Association (AVMA) professional codes and standards.

### Behavioural training and testing procedures

#### Preparation

Behavioural conditioning was done using a non-aversive appetitive classical conditioning protocol (modified from [26]; see also [32]). Snails were sampled at random and marked for identification purposes with indelible marker. Food was withheld starting 48 hrs prior to the first pre-training test and for the remainder of the training and post-training testing.

#### Testing procedures

On day 1, prior to behavioural conditioning, snails were individually tested for their natural response to the administration of pond water, the disturbance stimulus (DS), as well as the conditional stimulus (CS) n-amyl acetate (“Pre-training test”; see also [32]). Tests were performed using 100-ml translucent polystyrene beakers (4.5 cm diameter), filled with 80 ml of water taken from the snail’s home tank. After transfer into the beakers, the snails were allowed to acclimatize for 15 min before testing commenced. Testing involved counting the number of rasps over two consecutive periods of 2 min, the first period starting with gentle administration of the DS (10 ml artificial pond water), the second period starting with the administration of the CS (10 ml n-amyl acetate solution; 4 ppm final concentration). To facilitate observation, the test beakers were elevated on translucent plastic stands surrounded by mirrors. A test response was calculated by taking the difference between the number of rasps counted during the second period minus the number counted during the first period (i.e., ΔRasp = rasps after CS – rasps after DS). To correct for both differences in background rasping activity and potential application artefacts, the pre-training tests were performed in duplicate with >1 hr interval, and behavioural responses were calculated as the average of both ΔRasp (i.e., ΔRasp_pre-test_ = average ΔRasp_pre-test_1 and ΔRasp_pre-test_2). After completion of a test, snails were gently rinsed with clean pond water and returned to their home tanks. Following identical procedures as described for the pre-testing above, a single post-training test was performed 1-2 hrs after training to asses intermediate term memory (ITM) or on day 3 (i.e., 24 hrs after training) to asses long-term memory (LTM) performance.

#### Training procedure

Snails were trained in a single day, multi-trial forward-delay conditioning format (modified from [26]; see also [32]). Sucrose (final concentration of 0.4% wt/vol) served as the unconditioned stimulus (UCS) and n-amyl acetate (4 ppm final concentration) as the conditioned stimulus (CS). To control for potential behavioural effects of fluid addition, a disturbance control in which the UCS was paired with the DS (i.e., pond water) was implemented. Snails were randomly assigned to either the CS–UCS (“conditioned”) or the CS–DS (unconditioned “control”) group and trained “en masse”. Training was performed in 1-L polypropylene beakers containing 480 ml clean artificial pond water. After transfer into the training beakers, the snails were allowed to acclimatize for 60 min. Both “control” and “conditioned” groups received 120 ml of the CS solution, followed 15 s later by 120 ml of the UCS (“conditioned” group) or 120 ml of the DS (“control” group). After 2 min, the beakers containing the snails were drained and gently rinsed with clean pond water and the snails were readied for their next training trial by re-placing them in the 1-L polypropylene beakers holding 480 ml clean artificial pond water. After 11 min and 45 sec the training procedure was repeated. Snails received a total of 5 training procedures on a single day before being returned to their “home” tank. The snails were at all times fully submerged during training and testing. Care was taken to ensure that pre-training tests, training and post-training tests commenced at the same time of day for each group and training and testing always occurred in the same location. “Conditioned” and “control” snails were always tested and trained concurrently.

### Sucrose induced feeding behaviour in naïve animals

To assess whether laminarin induces chemosensory and/or motor deficiency, naïve snails injected with either laminarin or vehicle were tested for their feeding response upon sucrose application (see also [32]). Tests were performed using 100-ml translucent polystyrene beakers (4.5 cm diameter), filled with 80 mL of water taken from the snail’s home tank. After transfer into the beakers, the snails were allowed to acclimatize for 15 min before testing commenced. Testing involved counting the number of rasps over three consecutive periods of 2 min, the second period starting with gentle administration of artificial pond water (10 ml) a disturbance stimulus (DS), the third period starting with the administration of sucrose solution (0.4% w/v final concentration). The disturbance response was calculated by taking the difference between the number of rasps counted during the second period minus the number counted during the first period (i.e., ΔRasp disturbance = rasps after DS – rasps after pond water). The sucrose response was calculated by taking the difference between the number of rasps counted during the third period minus the number counted during the second period (i.e., ΔRasp sucrose = rasps after sucrose – rasps after DS).

### Electrophysiology

For electrophysiological assessments, snails were de-shelled using curved forceps and subsequently anaesthetized in a 25% Listerine solution. All dissections and experiments were carried out in a hydroxyethylpiperazine ethanesulphonic acid (HEPES) -buffered saline (HBS) composed of (in mM) 51.3 NaCl, 1.7 KCl, 4.1 CaCl_2_, 1.5 MgCl_2_ and 10 HEPES (pH 7.9).

#### Extracellular recordings

Extracellular recordings of lip sensory neurons were made from the superior lip nerve in a semi-intact preparation by means of a suction electrodes as described by Watson et al., [32]. Briefly, the central nervous system (CNS) was exposed by means of a small dorsal incision in the midline of the head region. The head of the snail containing the lips, tentacles and buccal mass, plus the CNS was carefully dissected from the rest of the body. Subsequently the right superior lip nerve was cut as close to the CNS as possible. The remaining peripheral nerves were cut as distally as possible and the CNS was discarded. The semi-intact preparation was pinned down in a Sylgard coated dish, and the superior lip nerve was drawn into a glass microelectrode with a diameter just large enough to accommodate the nerve. A perfusion system was used to continuous apply saline or n-amyl acetate (4 ppm) with the outflow placed close to the lip/mouth region. Extracellular signals were amplified using a DAM-80 differential amplifier (World Precision Instruments, Sarasota, FL), band-pass filtered at 10-1000 Hz and digitized at 5 kHz sampling rate using a Digidata 1322A AD/DA converter under the control of Axoscope 9 software (both Axon Instruments, Union City, CA). Recordings were analyzed offline using template-matching waveform recognition software (Spike2, version 4.02a, Cambridge, England). Template selection criteria were set to ignore waveforms that occurred less frequently than 1 in 50 events. From these data overall peristimulus histograms were generated with bin sizes of 30 seconds.

#### Intracellular recordings

CNSs were dissected as described earlier [69]. Isolated CNSs were pinned down in an elastomer covered recording chamber filled with HBS. To provide access to the Cerebral Giant Cells (CGCs), the outer layer of connective tissue surrounding the cerebral ganglia were removed with fine forceps without the use of proteolytic enzymes [69,70]. Intracellular recordings of the CGCs were done by means of microelectrode recording techniques with the use of either Axoclamp 2A or Axoclamp 2B, (Axon Instruments, Burlingame, CA). Amplifier output was filtered at 1 kHz and digitized at 5 kHz using a Digidata 1322A AD/DA converter (Axon Instruments, Burlingame, CA). Data acquisition was carried out with Axoscope sampling software (version 9.0, Axon Instruments) and subsequently analyzed using Clampfit software (version 9.0 Axon Instruments). Borisilicate glass electrodes (TW150F, World Precision Instruments, Sarasota, FL) were filled with a solution of 0.5 M potassium acetate (CH3COOK) and 0.01 M potassium chloride (KCl) saline. DC resistance of the electrodes ranged between 25-55 MΩ.

### Drug injections

Drug or vehicle-only treatments were delivered by means of intracoelomic injection into the snail in a manner avoiding whole-body withdrawal responses and voiding of haemolymph (see also [6,32]). Drugs were dissolved in sterilized ultrapure water or DMSO. Using body weight measurements injected amounts of drugs were calculated to achieve the following approximate concentrations of drugs in the snails’ haemolymph: 10 μM of the PLA_2_ inhibitor aristolochic acid, 5 mg/ml of laminarin, 10 μM of indomethacin and 10 μM of nordihydroguaiaretic acid (NDGA). Haemolymph DMSO concentrations never exceeded 0.02%. As control, snails were injected with equivalent volumes vehicle only (vehicle control). Unless indicated otherwise, all injections were given 1 hour prior to the start of the first training session. All solutions were injected through the foot directly into the haemocoel with a microliter syringe and a 25G needle. Snails were behaving normally within 1-2 minutes after injection.

### Haemocyte respiratory burst assays

#### *In vivo*

To quantify the effect of various *in vivo* treatments on haemocyte activity status, 200-300 μl samples of haemolymph per snail were taken 0.5 hr, 1 hr, 3 hrs or 24 hrs after injection using a non-invasive technique described by Sminia [71]. For each treatment condition haemolymph sampled from at least 3 snails was pooled, diluted in filtered sterilized HBS (1:2, HBS:haemolymph) and placed on ice (see also [36]). Hydrogen peroxide release by isolated haemocytes was measured using an Amplex Red assay according to the suppliers instructions (Invitrogen, Burlington, ON). Briefly, diluted haemolymph was added to a 96-well Nunc culture plate (50 μl per well) containing 150 μl Amplex red reaction solution (50 μM Amplex Red; reagent; 0.1 U/ml horseradish peroxidase). Immediately, fluorescence intensity was measured (544 nm excitation, 590 nm emission) using a Spectromax 2Me multidetection microplate reader (Molecular Devices, Sunnyvale, CA). Three independent experiments with each 3-4 replicates/condition were performed.

#### *In vitro*

The inhibitory effect of various compounds on haemocyte H_2_O_2_ production *in vitro* was determined according to a procedure described by Lacchini et al. [35]. Briefly haemolymph samples of 20 snails were collected, pooled, diluted in filter sterilized HBS (1:2, HBS:haemolymph) and stored on ice. Diluted haemolymph was added to a 96-well Nunc culture plate (200 μl per well). After a 30 minutes equilibration period, aristolichic acid (10 μM), indomethacin (10 μM), or vehicle only were added to the samples. After an additional 30 minutes, 100 μl Amplex red reaction solution (see above) plus laminarin (5 mg/ml) was added to each sample and incubated for an additional 30 minutes. Fluorescence intensity was measured as described above.

### Solutions and chemical suppliers

With the exception of the Amplex red hydrogen peroxide/peroxidase assay kit purchased from Invitrogen (Burlington, ON) and indomethacin obtained from Calbiochem (San Diego, CA), all chemicals were obtained from Sigma Aldrich (St. Louis, MO).

### Statistical analysis

Behavioural data was analyzed by means of fully-factorial or repeated measure ANOVA. Explicit hypotheses were tested using planned comparisons. Haemocyte hydrogen peroxide release data was analyzed by means of repeated measures ANOVA. Compliance with parametric assumptions was confirmed for all data sets prior to ANOVA using both graphical (probability plots applied to raw data and residuals) and analytical techniques (Levene’s test, Kolmogorov-Smirnov one-sample test for normality and F-max test). Throughout the text, average and data dispersion are expressed as arithmetic means and standard error of the mean (SEM). Figures were constructed using Graphpad Prism version 4.03 (Graphpad Software Inc., La Jolla, CA).

## Abbreviations

ROS: Reactive oxygen species; RNS: Reactive nitrogen species; LTM: Long-term memory; DS: Disturbance stimulus; CS: Conditioned stimulus; UCS: Unconditioned stimulus; PLA2: Phospholipase A_2_; HBS: HEPES buffered saline; RSLN: Right superior lip nerve; COX: Cyclooxygenase; LOX: Lipoxygenase; NDGA: Nordihydroguaiaretic acid; CNS: Central nervous system.

## Competing interests

The authors declare that they have no competing interests.

## Authors’ contributions

Conceived research program WCW. Conceived and designed the experiments: PMH, WCW. Performed the experiments: DP, EB, PMH. Analyzed the data DP, EB, PMH, WCW. Wrote the paper: PMH, WCW. All authors read and approved the final manuscript.
